# A Facile Ionic Liquid Promoted Synthesis, Cholinesterase Inhibitory Activity and Molecular Modeling Study of Novel Highly Functionalized Spiropyrrolidines

**DOI:** 10.3390/molecules20022296

**Published:** 2015-01-29

**Authors:** Abdulrahman I. Almansour, Raju Suresh Kumar, Natarajan Arumugam, Alireza Basiri, Yalda Kia, Mohamed Ashraf Ali, Mehvish Farooq, Vikneswaran Murugaiyah

**Affiliations:** 1 Department of Chemistry, College of Science, King Saud University, P.O. Box 2455, Riyadh 11451, Saudi Arabia; E-Mails: almansor@ksu.edu.sa (A.I.A.); aruorgchem@gmail.com (N.A.); 2 School of Pharmaceutical Sciences, Universiti Sains Malaysia, Minden 11800, Malaysia; E-Mails: diba115920@gmail.com (A.B.); vicky@usm.my (V.M.); 3 School of Chemical Sciences, Universiti Sains Malaysia, Minden 11800, Malaysia; E-Mail: kia.yalda@yahoo.com; 4 Pharmacogenetic and Novel Therapeutic Institute for Research in Molecular Medicine, Universiti Sains Malaysia, Minden 11800, Malaysia; E-Mail: asraf80med@yahoo.com; 5 Institute of Pharmaceutical Science, King’s College London, London SE1 9NH, UK; E-Mail: Mehvish.farooq@kcl.ac.uk

**Keywords:** acetylcholinesterase (AChE), butyrylcholinesterase (BChE), Alzheimer’s disease (AD), spiropyrrolidines, ionic liquid, molecular docking study

## Abstract

A series of novel dimethoxyindanone embedded spiropyrrolidines were synthesized in ionic liquid, [bmim]Br and were evaluated for their inhibitory activities towards cholinesterases. Among the spiropyrrolidines, compound **4f** exhibited the most potent activity with an IC_50_ value of 1.57 µM against acethylcholinesterase (AChE). Molecular docking simulation for the most active compound was employed with the aim of disclosing its binding mechanism to the active site of AChE receptor.

## 1. Introduction

Alzheimer’s disease (AD), the most common cause of dementia, is a neurodegenerative disorder characterized by progressive decline of memory and cognition [1]. One of the major therapeutic strategies adopted for primarily symptomatic AD is based on the cholinergic hypothesis targeting cholinesterase enzymes [2]. Inhibition of the hydrolysis of acetylcholine (ACh) by blocking its metabolic enzyme acetylcholinesterase (AChE) increases the ACh concentration and provides a possible symptomatic treatment option for AD. On the other hand, butyrylcholinesterase (BChE) has recently been considered as a potential target because it also plays an important role in regulating ACh level [3]. Although there are many ongoing research activities in the search of drugs for treating AD, only four drugs are currently approved for treatment and these drugs do not show potential cure rates. However, from academic and practical standpoints, it is still desirable to develop more effective agents for the treatment of AD. The increasing mortality rate and the reduced therapeutic potential of the currently available options have led us to focus our research activities on the development of potential cholinesterase inhibitors for the treatment of AD.

Among the five-membered heterocyclic systems, spiropyrrolidines occupy an important place as this structural motif appears as an integral part of many natural products besides exhibiting anticonvulsant [4,5,6], potential antileukemic [7], local anaesthetic [8] and antiviral activities [9]. Ionic liquids are widely recognized as “green” solvents in organic synthesis because of their unique properties, such as low vapor pressure, high chemical and thermal stability, solvating ability, non-flammability, behavior as acidic or basic catalysts and recyclability [10,11,12,13,14]. In this context, ionic liquids have emerged as new green solvents to replace the volatile organic compounds and they are found suitable for executing many diverse organic reactions [15,16,17]. The development of multi-component reactions in ionic liquids, although relatively unexplored [18,19,20,21,22] is of great interest and to the best of our knowledge, this is the first report on 1,3-dipolar cycloaddition reaction of 2-arylmethylidene-5,6-dimethoxy-2,3-dihydro-1*H*-inden-1-one with an unexplored and new class of azomethine ylide generated *in situ* from *L*-phenylalanine and isatin in ionic liquid medium, namely 1-butyl-3-methylimidazolium bromide ([bmim]Br). 

As a part of our ongoing research project aimed at identifying potential cholinesterase inhibitors for AD therapy [23,24,25,26,27,28,29,30,31,32,33], we report here our efforts in search of novel potent cholinesterase inhibitors using dipolar cycloaddition strategy. In addition, molecular modeling studies were also performed to disclose the binding modes of the most active inhibitors to the amino acid residues that compose the active site of the AChE enzyme.

## 2. Results and Discussion

### 2.1. Chemistry

**Scheme 1:**
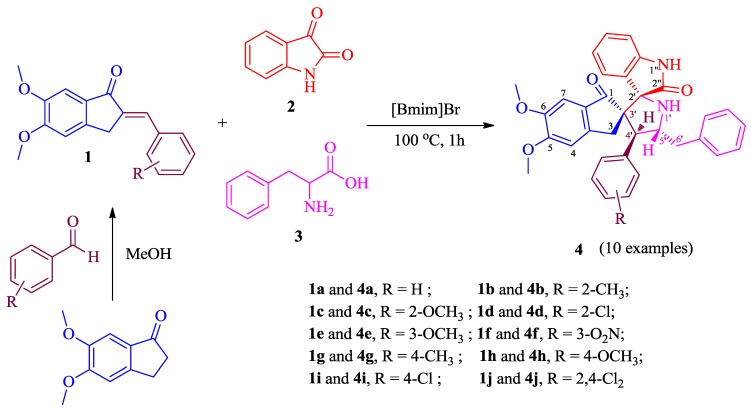
Synthesis of spiropyrrolidines **4a**–**j**.

The target spiropyrrolidines **4a**–**j** were synthesized as outlined in Scheme 1. The required starting precursors 2-arylmethylidene-5,6-dimethoxy-2,3-dihydro-1*H*-inden-1-ones **1a**–**j** were synthesized following the synthetic protocol reported earlier by us [34] by the reaction of 5,6-dimethoxy-1-indanone with appropriate aromatic aldehydes in dilute methanolic sodium hydroxide. The three component 1,3-dipolar cycloaddition reaction of a series of **1a**–**j** with a new class of relatively unexplored azomethine ylide generated *in situ* from isatin (**2**) and phenylalanine (**3**) in [bmim]Br afforded the functionalized spiropyrrolidines **4a**–**j** in good yields.

Solvent optimization for this cycloaddition reaction was investigated by choosing the model reaction between azomethine ylide and 2-(2-methylphenyl)methylidene-5,6-dimethoxy-2,3-dihydro-1*H*-inden-1-one (**1b**). The reaction of an equimolar mixture of **1b**, isatin and phenylalanine in refluxing methanol, methanol:dioxane and dioxane for 5 h afforded the spiropyrrolidine (**4b**) in 44%, 52% and 58% yield whilst the reaction in [bmim]Br furnished **4b** in an excellent yield of 87% in a short reaction time of 1 h, suggesting [bmim]Br as the appropriate solvent in terms of enhanced yield and reaction time for the cycloaddition reactions of this kind. Having determined the optimal conditions, all the subsequent reactions were effected by heating an equimolar mixture of the reactants in [bmim]Br (200 mg) in an oil-bath at 100 °C for 1 h. After completion of the reaction as evidenced by TLC, the product was isolated and purified through column chromatography, whilst the ionic liquid is recovered and reused, its efficacy being significantly undiminished in subsequent runs. As shown in Table 1, the cycloaddition reaction works well regardless of the position and electronic or steric properties of the substituents at the aromatic ring of compound **1** affording the cycloadduct **4** in good yields. 

The structure of spiropyrrolidines **4** was elucidated unambiguously with the help of one and two-dimensional NMR spectroscopic studies. As a representative case the chemical shift assignments of **4b** are discussed. In the ^1^H-NMR spectra of **4b**, the doublet at 4.10 ppm (*J* = 8.8 Hz) can be assigned to H-4'. H,H-COSY correlation of H-4' assigns the multiplet at 4.95–5.01 ppm to H-5'. The C,H-COSY correlation of H-4' and H-5' assigns the carbon signals at 55.44 and 64.85 ppm to C-4' and C-5', respectively. Further, the H,H-COSY correlation of H-5' assigns the doublet of doublets at 2.93 (*J* = 14.0, 8.8 Hz) and 3.02 (*J* = 14.0, 4.4 Hz) to the benzylic protons (6'-CH_2_). The C,H-COSY correlation of 6'-CH_2_ assigns the carbon signal at 40.45 ppm to C-6'. The remaining doublets at 2.62 and 2.73 ppm (*J* = 18.3 Hz) are due to 3-CH_2_. Further, H-4' shows HMBCs with the C=O of indanone ring, C-5', C-6', C-3 besides showing correlation with the aromatic ring carbons. The carbon signals at 66.69 and 75.08 ppm are due to the spiro carbons C-3' and C-2', respectively. The carbon chemical shifts of C-3, C-4', C-5' and C-6' were also confirmed from DEPT 135 NMR spectrum (vide supplementary Materials).The singlets at 1.92, 3.70, 3.80 and 8.01 ppm can be attributed to the methyl, two methoxy of aromatic rings and the NH group of oxindole ring, respectively. The aromatic protons appear as singlets, doublets and multiplets around 6.19–8.05 ppm. The signals at 179.63 and 207.10 ppm are due to the carbonyl groups of oxindole and indanone ring, respectively. The structure of other spiropyrrolidines was also assigned by similar straightforward considerations. The stereochemistry of compound **4b** has been arrived from its NOESY spectra.

**Table 1:** Physical data, AChE and BChE inhibitory activities of **4a**–**j**.

Entry	Comp 4	R	Yield (%) ^a^	mp °C	AChE Inhibition IC_50_ µM (±SD)	BChE Inhibition IC_50_ µM (±SD)
1	**a**	H	85	134–135	11.29 ± 0.21	15.73 ± 0.22
2	**b**	2-CH_3_	87	141–142	10.15 ± 0.17	21.32 ± 0.19
3	**c**	2-OCH_3_	82	126–127	12.23 ± 0.19	11.39 ± 0.12
4	**d**	2-Cl	85	136–137	15.17 ± 0.22	9.63 ± 0.15
5	**e**	3-OCH_3_	84	127–128	2.13 ± 0.11	17.44 ± 0.23
6	**f**	3-O_2_N	89	133–134	1.57 ± 0.09	15.32 ± 0.18
7	**g**	4-CH_3_	92	138–139	7.11 ± 0.13	10.46 ± 0.24
8	**h**	4-OCH_3_	80	129–130	3.67 ± 0.15	14.69 ± 0.17
9	**i**	4-Cl	88	132–133	5.83 ± 0.20	16.11 ± 0.15
10	**j**	2,4-Cl_2_	90	135–136	6.72 ± 0.18	12.79 ± 0.25
11	Galantamine	-	-	-	2.09 ± 0.07	19.21 ± 0.12
12	Donepezil	-	-	-	0.21 ± 0.02	3.6 ± 0.11

^a^ Isolated yield.

**Scheme 2:**
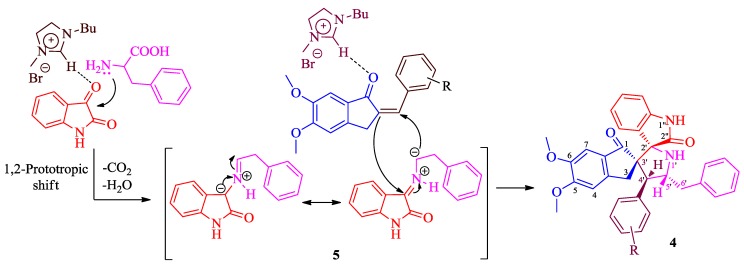
Plausible mechanism for the formation of spiropyrrolidine **4**.

A mechanism proposed to rationalize the formation of **4** is summarized in Scheme 2. Hydrogen atom of [bmim]^+^ being electron-deficient could form hydrogen bond between the imidazole ring hydrogen atom of [bmim]^+^ and the carbonyl group of isatin facilitating the attack of lone pair of phenylalanine on isatin and subsequent dehydration to furnish azomethine ylide **5**. In the same way, hydrogen atom of [bmim]^+^ forms hydrogen bond with the carbonyl group of 2-arylmethylidene-5,6-dimethoxy-2,3-dihydro-1*H*-inden-1-one providing the easy addition of azomethine ylide to the more electron deficient β-carbon to afford spiropyrrolidine **4**, regio- and stereoselectively.

**Scheme 3:**
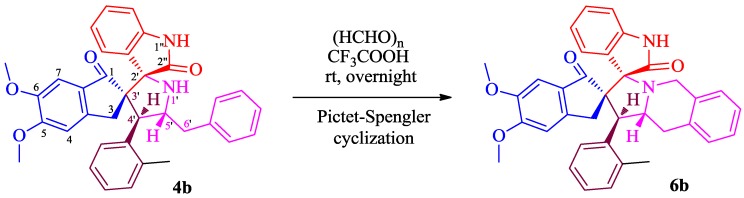
Pictet-Spengler cyclisation of spiropyrrolidine **4b**.

The presence of NH and benzyl groups in adjacent positions of the pyrrolidine ring of **4** prompted us to explore their further reaction with paraformaldehyde (Pictet–Spengler cyclisation) in the presence of trifluoroacetic acid to synthesize novel isoquinoline heterocyclic hybrids. Thus, the spiropyrrolidine (**4b**) (1 mmol) was treated overnight with paraformaldehyde (1 mmol) in the presence of a catalytic amount of trifluoroacetic acid (10 mol %) in CH_2_Cl_2_ at room temperature (Scheme 3). This reaction afforded the mixture of products with same R_f_ value, which could not be separated through column chromatography.

### 2.2. Cholinesterase Inhibitory Activity Study

All newly synthesized compounds were evaluated *in vitro* for their cholinesterase inhibitory activity against AChE enzyme from electric eel and BChE enzyme from equine serum. As summarized in Table 1, compound **4f**, bearing nitro moiety at meta position of aromatic ring displayed highest AChE inhibitory activity with calculated IC_50_ value of 1.57 µM, followed by **4e** (Ar=*m*-OCH_3_), **4h** (Ar=*p*-OCH_3_), **4i** (Ar=*p*-Cl), **4j** (Ar=*o,p*-Cl_2_) and **4g** (Ar=*p*-CH_3_) all displaying remarkable IC_50_ values of lower than 10 µM. The rest of the compounds **4a**–**d** displayed moderate AChE inhibitory activities ranging from 10.15–15.17 µM. From Table 1, it is clear that the presence of electron withdrawing nitro moiety, had greater impact on the AChE inhibitory potencies compared to electron donating moieties such as methoxy (strong electron donor), methyl and chloro (mild electron donor).

The active site of AChE enzyme is located at bottom of a narrow channel composed of amino acid residues with aromatic side chains such as tryptophan and tyrosine. The potency of inhibitors is closely related to the ease of insertion into this channel and their ability to interact with its composing residues. In fact, inhibitors having more facilitated insertion and better interactions with this gorge should possess higher enzyme inhibitory potentials. Thus, the inhibitor 4f, owing to the unique electronic withdrawing nature of its nitro group, strongly binds to the AChE active site and effectively inhibited choline substrate hydrolysis. Interestingly, compounds bearing a strong electron donor methoxy moiety, also showed good inhibitory activities but at much lower degree when compared to those containing a nitro group.

It is also worth to mention that these newly compounds are benefited from their specific chemical structure of including three aromatic cores, by showing moderate to weak BChE inhibitory potencies with calculated IC_50_ values ranging from 9.63–21.32 µM and good selectivity toward AChE enzyme. The aromatic core multiplicity effect explains how compounds bearing three aromatic cores have higher AChE inhibitory potencies than their analogs with only two aromatic cores and introduces the aromatic content of inhibitor as an important factor to design potent AChE inhibitors [28].

Compared to standard drugs, galantamine and donepezil, compound **4f** displayed significantly higher and compound **4e** showed comparable AChE inhibitory activity to galantamine. None of the inhibitors had comparable activity to donepezil, due to its different planar chemical structure. For BChE, except for compound **4b**, all the inhibitors showed higher activities than galantamine. As expected, none of the compounds showed comparable BChE activity to donepezil.

### 2.3. Molecular Docking Studies

**Figure 1:**
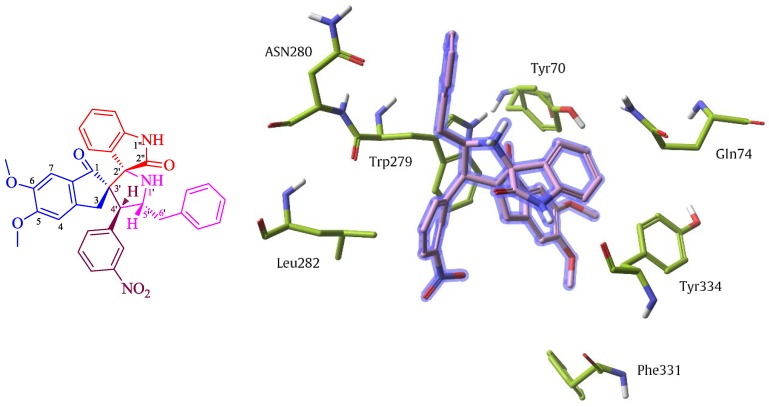
Schematic representation of compound **4f** bounded to active site of AChE enzyme.

The most active AChE inhibitor, compound **4f** was docked into the active site of AChE enzyme derived from the crystal structure of *Torpedo californica* in complex with donepezil (Figure 1). Molecular docking analysis clearly demonstrated the influence of aromatic core multiplicity in the remarkable activity observed for this compound. Compound **4f** strongly bounded to the residues having aromatic side chains such as Trp279, Tyr70, Tyr334 and Phe331, which compose peripheral anionic site (PAS) of the AChE enzyme through hydrophobic interactions took place dominantly between their aromatic entities. It was proposed that deposition of neurotoxin amyloid plaque in AD could be accelerated or even triggered by interaction of β-amyloids with the peripheral anionic site of the enzyme. Therefore, PAS inhibitors not only symptomatically improve the AD by blocking active site gorge entrance and prevention of acetylcholine hydrolysis, but also can have effective disease modifying properties [35].

## 3. Experimental Section

### 3.1. General Methods

Melting points were measured using open capillary tubes and are uncorrected. ^1^H- and ^13^C-NMR spectra were recorded on Jeol 500 and 400 MHz instruments (Tokyo, Japan) in CDCl_3_ using TMS as internal standard. Chemical shifts are given in parts per million (δ scale) and coupling constants are given in Hertz. IR spectra were recorded on a PerkinElmer spectrum 100 FT-IR instrument (KBr pellet, Shelton, AL, USA). Elemental analyses were performed 10 on a PerkinElmer 2400 Series II Elemental CHNS analyzer (Waltham, MA, USA). Column chromatography was performed on silica gel (230–400 mesh) using petroleum spirit/ethyl acetate as eluent.

### 3.2. General Procedure for the Synthesis of ***4***

An equimolar mixture of 2-arylmethylidene-5,6-dimethoxy-2,3-dihydro-1*H*-inden-1-one (**1**), isatin (**2**) and phenylalanine (**3**) was heated with stirring at 100 °C in 200 mg of [bmim]Br for 1 h. After completion of the reaction (TLC), ethyl acetate (10 mL) was added and the reaction mixture stirred for 15 min. The ethyl acetate layer was separated, washed with water and dried. The product obtained in good yield was purified through column chromatography. [bmim]Br after extraction of the product was completely dried under vacuum and reused for subsequent reactions.

### 3.3. Characterization Data for Compounds ***4a***–***j***

*5'-Benzyl-5,6-dimethoxy-2,3-dihydro-4'-phenyldispiro[1H-indene-2,3'-pyrrolidine-2',3''-indoline]-1,2''-dione* (**4a**). IR (KBr) ν_max_ 3405, 1721, 1685, 1620, 1594 cm^−1^; ^1^H-NMR (400 MHz, CDCl_3_): δ_H_ 2.65 (d, *J* = 17.6 Hz, 1H, 3-CH_2_), 2.86–2.97 (m, 2H, 3-CH_2_ and 6'-CH_2_), 3.04–3.08 (m, 1H, 6'-CH_2_), 3.65 (s, 3H, OCH_3_), 3.78 (s, 3H, OCH_3_), 3.94 (d, *J* = 8.8 Hz, 1H, H-4'), 4.70–4.81 (m, 1H, H-5'), 6.19 (s, 1H, Ar-H), 6.58 (d, *J* = 7.2 Hz, 1H, Ar-H), 6.86–7.40 (m, 14H, Ar-H), 8.37 (s, 1H, NH). ^13^C-NMR (100 MHz, CDCl_3_): δ_C_ 34.27 (C-3), 40.10 (C-6'), 56.09 (OCH_3_), 56.15 (OCH_3_), 59.47 (C-4'), 64.00 (C-5'), 67.16 (C-3'), 74.96 (C-2'), 104.08, 106.49, 109.63, 122.52, 126.37, 128.30, 128.45, 129.19, 129.27, 129.40, 129.62, 129.74, 130.21, 135.48, 136.57, 138.85, 148.17, 149.42, 155.44, 180.13 (C-2''), 206.35 (C-1). Anal. calcd for C_34_H_30_N_2_O_4_: C, 76.96; H, 5.70; N, 5.28. Found: C, 76.82; H, 5.47; N, 5.19%.

*5'-Benzyl-5,6-dimethoxy-2,3-dihydro-4'-(2-methylphenyl)dispiro[1H-indene-2,3'-pyrrolidine-2',3''-indoline]-1,2''-dione* (**4b**). IR (KBr) ν_max_ 3427, 1720, 1687, 1619, 1591 cm^−1^; ^1^H-NMR (400 MHz, CDCl3): δ_H_ 1.92 (s, 3H, CH_3_), 2.62 (d, *J* = 18.32 Hz, 1H, 3-CH_2_), 2.73 (d, *J* = 18.32 Hz, 1H, 3-CH_2_), 2.93 (dd, *J* = 13.96, 8.0 Hz, 1H, 6'-CH_2_), 3.02 (dd, *J* = 13.92, 4.4 Hz, 1H, 6'-CH_2_), 3.70 (s, 3H, OCH_3_), 3.80 (s, 3H, OCH_3_), 4.10 (d, *J* = 8.8 Hz, 1H, H-4'), 4.95–5.01 (m, 1H, H-5'), 6.19 (s, 1H, Ar-H), 6.57 (d, *J* = 7.32 Hz, 1H, Ar-H), 6.86 (t, *J* = 7.32 Hz, 1H, Ar-H), 6.90 (s, 1H, ArH), 6.99–7.31 (m, 9H, Ar-H), 7.36 (d, *J* = 7.36 Hz, 1H, Ar-H), 8.01 (s, 1H, NH), 8.04 (d, *J* = 8.0 Hz, 1H, Ar-H). ^13^C-NMR (100 MHz, CDCl_3_): δ_C_ 20.33 (CH_3_), 35.16 (C-3), 40.45 (C-6'), 55.44 (C-4'), 55.95 (OCH_3_), 56.04 (OCH_3_), 64.85 (C-5'), 66.69 (C-3'), 75.08 (C-2'), 103.96, 106.41, 109.35, 122.51, 126.13, 126.23, 126.51, 126.69, 127.05, 127.71, 128.23, 129.00, 129.14, 129.66, 130.08, 137.46, 137.91, 138.92, 140.92, 148.09, 149.27, 155.36, 179.63 (C-2''), 207.10 (C-1). Anal. calcd for C_35_H_32_N_2_O_4_: C, 77.18; H, 5.92; N, 5.14. Found: C, 77.39; H, 5.78; N, 5.02%.

*5'-Benzyl-5,6-dimethoxy-2,3-dihydro-4'-(2-methoxyphenyl)dispiro[1H-indene-2,3'-pyrrolidine-2',3''-indoline]-1,2''-dione* (**4c**). IR (KBr) ν_max_ 3429, 1722, 1688, 1621, 1595 cm^−1^; ^1^H-NMR (400 MHz, CDCl_3_): δ_H_ 2.40–2.54 (m, 2H, 3-CH_2_), 2.91–3.09 (m, 2H, 6′-CH_2_), 3.66 (s, 3H, OCH_3_), 3.69 (s, 3H, OCH_3_) 3.87 (s, 3H, OCH_3_), 4.14 (d, *J* = 9.2 Hz, 1H, H-4'), 5.02–5.10 (m, 1H, H-5'), 6.10 (s, 1H, Ar-H), 6.53 (d, *J* = 7.2 Hz, 1H, Ar-H), 6.61 (d, *J* = 8.0 Hz, 1H, Ar-H), 6.82–7.40 (m, 11H, Ar-H), 7.80 (d, *J* = 7.2 Hz, 1H, Ar-H), 8.59 (s, 1H, NH). ^13^C-NMR (100 MHz, CDCl_3_): δ_C_ 34.72 (C-3), 41.02 (C-6'), 53.48 (C-4'), 54.41 (OCH_3_), 56.13 (OCH_3_), 61.68 (OCH_3_), 65.53 (C-5'), 67.15 (C-3'), 75.01 (C-2'), 104.11, 106.40, 109.52, 109.79, 120.65, 122.59, 126.16, 126.67, 127.71, 128.15, 128.30, 128.98, 129.15, 129.25, 135.42, 139.34, 148.72, 149.09, 155.06, 158.17, 179.63 (C-2''), 208.06 (C-1). Anal. calcd for C_35_H_32_N_2_O_5_: C, 74.98; H, 5.75; N, 5.00. Found: C, 75.21; H, 5.61; N, 5.12%.

*5'-Benzyl-5,6-dimethoxy-2,3-dihydro-4'-(2-chlorophenyl)dispiro[1H-indene-2,3'-pyrrolidine-2',3''-indoline]-1,2''-dione* (**4d**). IR (KBr) ν_max_ 3422, 1716, 1684, 1620, 1595 cm^−1^; ^1^H-NMR (400 MHz, CDCl_3_): δ_H_ 2.51 (d, *J* = 18.0 Hz, 1H, 3-CH_2_), 2.64 (d, *J* = 18.0 Hz, 1H, 3-CH_2_), 2.96–3.10 (m, 2H, 6'-CH_2_), 3.69 (s, 3H, OCH_3_), 3.81 (s, 3H, OCH_3_), 4.39 (d, *J* = 8.8 Hz, 1H, H-4'), 4.92–4.99 (m, 1H, H-5′), 6.30 (s, 1H, Ar-H), 6.60 (d, *J* = 7.6 Hz, 1H, Ar-H), 6.92–7.47 (m, 10H, Ar-H), 7.95 (s, 1H, ArH), 8.07 (d, *J* = 7.6 Hz, 1H, Ar-H), 8.36 (s, 1H, NH). ^13^C-NMR (100 MHz, CDCl_3_): δ_C_ 34.77 (C-3), 40.78 (C-6'), 56.28 (OCH_3_), 56.39 (OCH_3_), 61.10 (C-4'), 66.28 (C-5'), 67.16 (C-3'), 75.37 (C-2'), 105.27, 106.54, 107.18, 109.66, 122.72, 126.81, 127.95, 128.26, 128.56, 129.08, 129.18, 129.84, 131.11, 133.87, 136.13, 137.39, 138.53, 149.39, 149.78, 155.45, 179.60 (C-2''), 207.11 (C-1). Anal. calcd for C_34_H_29_ClN_2_O_4_: C, 72.27; H, 5.17; N, 4.96. Found: C, 72.06; H, 5.41; N, 4.87%.

*5'-Benzyl-5,6-dimethoxy-2,3-dihydro-4'-(3-methoxyphenyl)dispiro[1H-indene-2,3'-pyrrolidine-2',3''-indoline]-1,2''-dione* (**4e**). IR (KBr) ν_max_ 3438, 1716, 1692, 1618, 1587 cm^−1^; ^1^H-NMR (400 MHz, CDCl_3_): δ_H_ 2.69 (d, *J* = 17.6 Hz, 1H, 3-CH_2_), 2.88–3.10 (m, 3H, 3-CH_2_ and 6'-CH_2_), 3.67 (s, 3H, OCH_3_), 3.72 (s, 3H, OCH_3_) 3.77 (s, 3H, OCH_3_), 4.08 (d, *J* = 8.8 Hz, 1H, H-4'), 4.75–4.82 (m, 1H, H-5'), 6.22 (s, 1H, Ar-H), 6.60 (d, *J* = 8.0 Hz, 1H, Ar-H), 6.71–7.28 (m, 13H, Ar-H), 8.72 (s, 1H, NH). ^13^C-NMR (100 MHz, CDCl_3_): δ_C_ 34.42 (C-3), 40.28 (C-6'), 55.22 (OCH_3_), 56.02 (OCH_3_), 56.14 (OCH_3_), 60.00 (C-4'), 64.26 (C-5'), 67.14 (C-3'), 74.94 (C-2'), 104.10, 106.65, 109.71, 112.54, 115.79, 122.53, 122.74, 123.17, 126.31, 128.10, 128.40, 129.21, 129.36, 129.40, 138.79, 140.45, 148.29, 149.32, 155.52, 159.68, 180.27 (C-2''), 206.44 (C-1). Anal. calcd for C_35_H_32_N_2_O_5_: C, 74.98; H, 5.75; N, 5.00. Found: C, 75.10; H, 5.86; N, 5.08%.

*5'-Benzyl-5,6-dimethoxy-2,3-dihydro-4'-(3-nitrophenyl)dispiro[1H-indene-2,3'-pyrrolidine-2',3''-indoline]-1,2''-dione* (**4f**). IR (KBr) ν_max_ 3425, 1721, 1684, 1623, 1590 cm^−1^; ^1^H-NMR (400 MHz, CDCl_3_): δ_H_ 2.55 (d, *J* = 17.6 Hz, 1H, 3-CH_2_), 2.90 (d, *J* = 17.6 Hz, 1H, 3-CH_2_), 2.94–2.98 (m, 1H, 6'-CH_2_), 3.10–3.19 (m, 1H, 6'-CH_2_), 3.73 (s, 3H, OCH_3_), 3.81 (s, 3H, OCH_3_), 3.88 (d, *J* = 8.8 Hz, 1H, H-4′), 4.78–4.84 (m, 1H, H-5'), 6.39 (s, 1H, Ar-H), 6.68 (d, *J* = 7.2 Hz, 1H, Ar-H), 6.85–7.86 (m, 10H, Ar-H), 7.67 (d, *J* = 7.2 Hz, 1H, Ar-H), 7.97 (d, *J* = 8.0 Hz, 1H, Ar-H), 8.28 (s, 1H, ArH), 8.47 (s, 1H, NH). ^13^C-NMR (100 MHz, CDCl_3_): δ_C_ 34.52 (C-3), 40.76 (C-6'), 56.12 (OCH_3_), 56.18 (OCH_3_), 60.09 (C-4'), 65.47 (C-5'), 67.16 (C-3'), 75.15 (C-2'), 104.32, 106.69, 109.70, 122.84, 122.98, 126.41, 126.53, 126.78, 127.82, 128.36, 129.18, 129.27, 136.75, 137.93, 141.00, 141.72, 147.74, 148.29, 149.66, 155.90, 179.76 (C-2''), 206.80 (C-1). Anal. calcd for C_34_H_29_N_3_O_6_: C, 70.94; H, 5.08; N, 7.30. Found: C, 70.68; H, 5.25; N, 7.41%.

*5'-Benzyl-5,6-dimethoxy-2,3-dihydro-4'-(4-methylphenyl)dispiro[1H-indene-2,3'-pyrrolidine-2',3''-indoline]-1,2''-dione* (**4g**). IR (KBr) ν_max_ 3420, 1723, 1691, 1620, 1589 cm^−1^; ^1^H-NMR (400 MHz, CDCl_3_): δ_H_ 2.30 (s, 3H, CH_3_), 2.67 (d, *J* = 17.6 Hz, 1H, 3-CH_2_), 2.85–2.94 (m, 2H, 3-CH_2_ and 6'-CH_2_), 3.07 (dd, *J* = 14.0, 2.8 Hz, 1H, 6'-CH_2_), 3.66 (s, 3H, OCH_3_), 3.77 (s, 3H, OCH_3_), 3.81 (d, *J* = 10.4 Hz, 1H, H-4'), 4.75–4.82 (m, 1H, H-5'), 6.14 (s, 1H, Ar-H), 6.56 (d, *J* = 8.0 Hz, 1H, Ar-H), 6.83–7.29 (m, 13H, Ar-H), 8.48 (s, 1H, NH). ^13^C-NMR (100 MHz, CDCl_3_): δ_C_ 21.15 (CH_3_), 34.24 (C-3), 40.13 (C-6'), 56.01 (OCH_3_), 56.14 (OCH_3_), 59.44 (C-4'), 63.92 (C-5'), 67.21 (C-3'), 74.84 (C-2'), 104.07, 106.61, 109.65, 122.54, 126.31, 128.22, 128.43, 129.14, 129.26, 129.37, 129.60, 129.75, 130.15, 135.45, 136.62, 138.87, 148.16, 149.30, 155.46, 180.26 (C-2''), 206.25 (C-1). Anal. calcd for C_35_H_32_N_2_O_4_: C, 77.18; H, 5.92; N, 5.14. Found: C, 77.43; H, 5.74; N, 5.01%.

*5'-Benzyl-5,6-dimethoxy-2,3-dihydro-4'-(4-methoxyphenyl)dispiro[1H-indene-2,3'-pyrrolidine-2',3''-indoline]-1,2''-dione* (**4h**). IR (KBr) ν_max_ 3426, 1722, 1691, 1622, 1596 cm^−1^; ^1^H-NMR (400 MHz, CDCl_3_): δ_H_ 2.69 (d, *J* = 17.6 Hz, 1H, 3-CH_2_), 2.85–2.96 (m, 2H, 3-CH_2_ and 6'-CH_2_), 3.03–3.08 (m, 1H, 6'-CH_2_), 3.71 (s, 3H, OCH_3_), 3.76 (s, 3H, OCH_3_), 3.78 (s, 3H, OCH_3_), 3.85–3.98 (m, 1H, H-4'), 3.67–3.75 (m, 1H, H-5'), 6.29 (s, 1H, Ar-H), 6.57 (d, *J* = 7.2 Hz, 1H, Ar-H), 6.79–7.62 (m, 13H, Ar-H), 8.09 (s, 1H, NH). ^13^C-NMR (100 MHz, CDCl_3_): δ_C_ 34.15 (C-3), 40.22 (C-6'), 55.27 (OCH_3_), 56.04 (OCH_3_), 56.15 (OCH_3_), 59.16 (C-4'), 64.12 (C-5'), 67.26 (C-3'), 74.73 (C-2'), 104.13, 106.70, 109.51, 113.88, 122.58, 126.31, 128.30, 128.42, 129.15, 129.35, 130.58, 131.31, 132.42, 138.89, 148.18, 149.31, 155.49, 158.55, 180.01 (C-2''), 206.30 (C-1). Anal. calcd for C_35_H_32_N_2_O_5_: C, 74.98; H, 5.75; N, 5.00. Found: C, 74.70; H, 5.96; N, 5.13%.

*5'-Benzyl-5,6-dimethoxy-2,3-dihydro-4'-(4-chlorophenyl)dispiro[1H-indene-2,3'-pyrrolidine-2',3''-indoline]-1,2''-dione* (**4i**). IR (KBr) ν_max_ 3389, 1718, 1685, 1615, 1594 cm^−1^; ^1^H-NMR (400 MHz, CDCl_3_): δ_H_ 2.68 (d, *J* = 17.6 Hz, 1H, 3-CH_2_), 2.89–2.99 (m, 2H, 3-CH_2_ and 6'-CH_2_), 3.06–3.10 (m, 1H, 6'-CH_2_), 3.68 (s, 3H, OCH_3_), 3.81 (s, 3H, OCH_3_), 3.95 (d, *J* = 8.8 Hz, 1H, H-4'), 4.72–4.84 (m, 1H, H-5'), 6.21 (s, 1H, Ar-H), 6.57 (d, *J* = 7.6 Hz, 1H, Ar-H), 6.83–7.45 (m, 13H, Ar-H), 8.40 (s, 1H, NH). ^13^C-NMR (100 MHz, CDCl_3_): δ_C_ 34.30 (C-3), 40.17 (C-6'), 56.10 (OCH_3_), 56.22 (OCH_3_), 59.45 (C-4'), 64.04 (C-5'), 67.13 (C-3'), 75.05 (C-2'), 104.12, 106.45, 109.62, 122.47, 126.46, 128.35, 128.43, 129.24, 129.31, 129.47, 129.61, 129.80, 130.29, 135.52, 136.61, 138.87, 148.20, 149.46, 155.49, 180.21 (C-2''), 206.63 (C-1). Anal. calcd for C_34_H_29_ClN_2_O_4_: C, 72.27; H, 5.17; N, 4.96. Found: C, 72.50; H, 5.02; N, 4.87%.

*5'-Benzyl-5,6-dimethoxy-2,3-dihydro-4'-(2,4-dichlorophenyl)dispiro[1H-indene-2,3'-pyrrolidine-2',3''-indoline]-1,2''-dione* (**4j**). IR (KBr) ν_max_ 3394, 1721, 1682, 1614, 1583 cm^−1^; ^1^H-NMR (400 MHz, CDCl_3_): δ_H_ 2.47 (d, *J* = 18.0 Hz, 1H, 3-CH_2_), 2.62 (d, *J* = 18.0 Hz, 1H, 3-CH_2_), 2.93 (dd, *J* = 14.0, 5.8 Hz, 1H, 6'-CH_2_), 2.99 (dd, *J* = 14.0, 5.8 Hz, 1H, 6'-CH_2_), 3.72 (s, 3H, OCH_3_), 3.81 (s, 3H, OCH_3_), 4.31 (d, *J* = 8.8 Hz, 1H, H-4'), 4.84–4.90 (m, 1H, H-5'), 6.30 (s, 1H, Ar-H), 6.59 (d, *J* = 7.2, 1H, Ar-H), 6.84–7.35 (m, 9H, Ar-H), 7.47 (s, 1H, ArH), 7.60 (d, *J* = 8.8 Hz, 1H, Ar-H), 8.00 (d, *J* = 8.8 Hz, 1H, Ar-H), 8.40 (s, 1H, NH). ^13^C-NMR (100 MHz, CDCl_3_): δ_C_ 34.66 (C-3), 40.88 (C-6'), 55.67 (OCH_3_), 56.08 (OCH_3_), 64.73 (C-4'), 66.06 (C-5'), 67.15 (C-3'), 75.37 (C-2'), 104.27, 106.55, 109.70, 122.81, 126.32, 126.51, 127.17, 127.83, 128.30, 128.73, 129.11, 129.53, 132.19, 132.79, 136.16, 136.60, 138.27, 141.23, 144.96, 148.02, 149.51, 155.59, 179.31 (C-2''), 206.98 (C-1). Anal. calcd for C_34_H_28_Cl_2_N_2_O_4_: C, 68.12; H, 4.71; N, 4.67. Found: C, 68.30; H, 4.94; N, 4.56%.

### 3.4. General Procedure for the Synthesis of ***6***

To a stirred solution of **4b** (1 mmol) in 5 mL of dry dichloromethane was added paraformaldehyde (1 mmol), followed by catalytic amount of trifluoroacetic acid at 0 °C. The reaction mixture was allowed to stir overnight and after completion of the reaction, the mixture was washed with water and dried over Na_2_SO_4_. The solvent was removed under reduced pressure to afford **6b** as mixture of products with same R_f_ value, which could not be separated through column chromatography.

### 3.5. Cholinesterase Inhibition Assays

Cholinesterase inhibitory activity of the synthesized compounds was evaluated using the Ellman’s microplate assay. For acetylcholinesterase (AChE) inhibitory assay, 140 μL of 0.1 M sodium phosphate buffer (pH 8) was first added to a 96-wells microplate followed by 20 μL of test samples and 20 μL of 0.09 units/mL acetylcholinesterase enzyme from *Electrophoruselectricus* (Sigma; St. Louis, MO, USA). After 15 min of incubation at 25 °C, 10 μL of 10 mM 5,5'-Dithiobis-2-nitrobenzoic acid (DTNB) was added into each well, followed by 10 μL of acetylthiocholine iodide (14 mM). At 30 min after the initiation of enzymatic reaction, absorbance of the colored end product was measured using BioTek Power Wave X 340 Microplate Spectrophotometer (BioTek; Winooski, VT, USA) at 412 nm. For butyrylcholinesterase (BChE) inhibitory assay, the same procedure described above was followed, except for the use of enzyme and substrate, instead of which, butyrylcholine esterase from equine serum and *S*-butyrylthiocholine chloride were used. Galanthamine was used as positive control. Test samples and galanthamine were prepared in DMSO at an initial concentration of 1 mg/mL (1000 ppm). The concentration of DMSO in final reaction mixture was 1%. At this concentration, DMSO has no inhibitory effect on acetylcholinesterase enzyme.

The initial screening was carried out at 10 µg/mL of test samples in 1% DMSO and each test was conducted in triplicates. Absorbance of the test samples was corrected by subtracting the absorbance of their respective blank. Percentage enzyme inhibition is calculated using the following formula:

(1) $$\text{Percentage of inhibition}=\;\frac{\left(\text{Absorbance of sample}\;-\;\text{Absorbance of control}\right)}{\text{Absorbance of control}\;\times 100}$$

Subsequently, the determination of IC_50_ was carried out using a set of five concentrations.

### 3.6. Molecular Modeling

Using Glide^TM^, (version 5.7, Schrödinger, LLC, New York, NY, USA, 2011), most active compound was docked onto the active site of *Tc*AChE derived from three-dimensional structure of the enzyme complex with anti-Alzheimer’s drug, galanthamine (PDB ID: 4EVE).

Water molecules and hetero groups were deleted from enzyme beyond the radius of 5 Å of reference ligand (galanthamine), resulting protein structure refined and minimized by Protein Preparation Wizard^TM^ (Version 2014-1, Schrödinger, LLC, New York, NY, USA) using OPLS-2005 force field. Receptor Grid Generation program were used to prepare *Tc*AChE grid and the ligand was optimized by LigPrep^TM^ (Version 2014-1, Schrödinger LLC, New York, NY, USA) program by using OPLS-2005 force field to generate lowest energy state. Docking stimulations were carried out on bioactive compound, handed in 5 poses per ligand, in which the best pose with highest score was displayed for each ligand.

## 4. Conclusions

In conclusion, the synthesis of a series of novel spiropyrrolidines was accomplished via [bmim]Br mediated one-pot three component 1,3-dipolar cycloaddition strategy employing a new class of azomethine ylide. Among the spiropyrrolidines **4a**–**j**, compound **4f** displayed the highest AChE enzyme inhibitory activity, Molecular-docking analysis, disclosed binding interactions of this compound to the active site residues of the AChE enzyme.

## Supplementary Materials

Copy of FT-IR, ^1^H and ^13^C spectra of a representative compound is available on supplementary file. Supplementary materials can be accessed at: http://www.mdpi.com/1420-3049/20/02/2296/s1.
