# Supplementary material for: A Facile Ionic Liquid Promoted Synthesis, Cholinesterase Inhibitory Activity and Molecular Modeling Study of Novel Highly Functionalized Spiropyrrolidines
Source: Molecules. 2015 Jan 29;20(2):2296–309. doi: 10.3390/molecules20022296 (PMC6272427; doi:10.3390/molecules20022296)
Supplement: Supplementary file 1 [file molecules-20-02296-s001.pdf]

Supplementary Materials

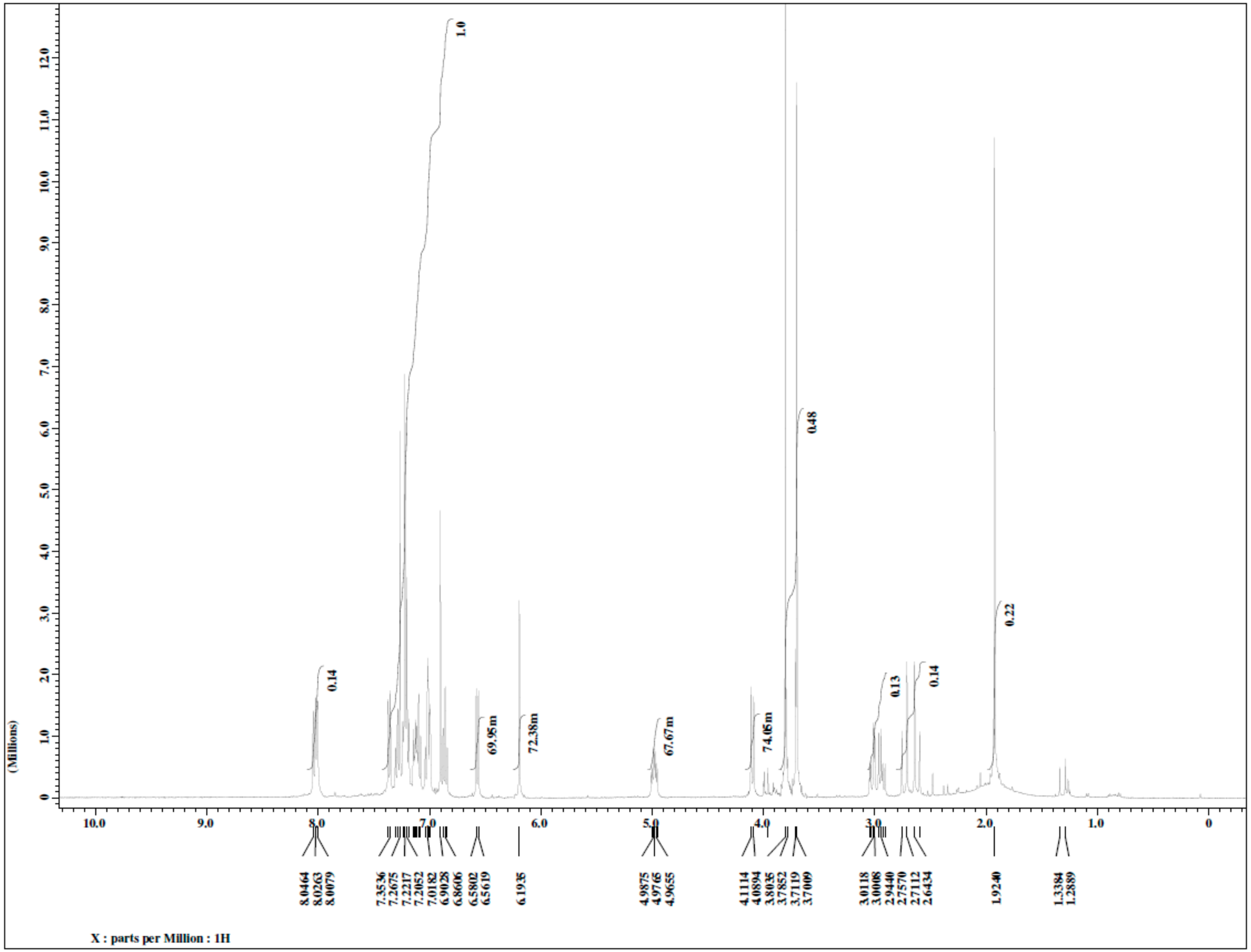

Figure S1. ^{1}H-NMR spectrum of **4b**.

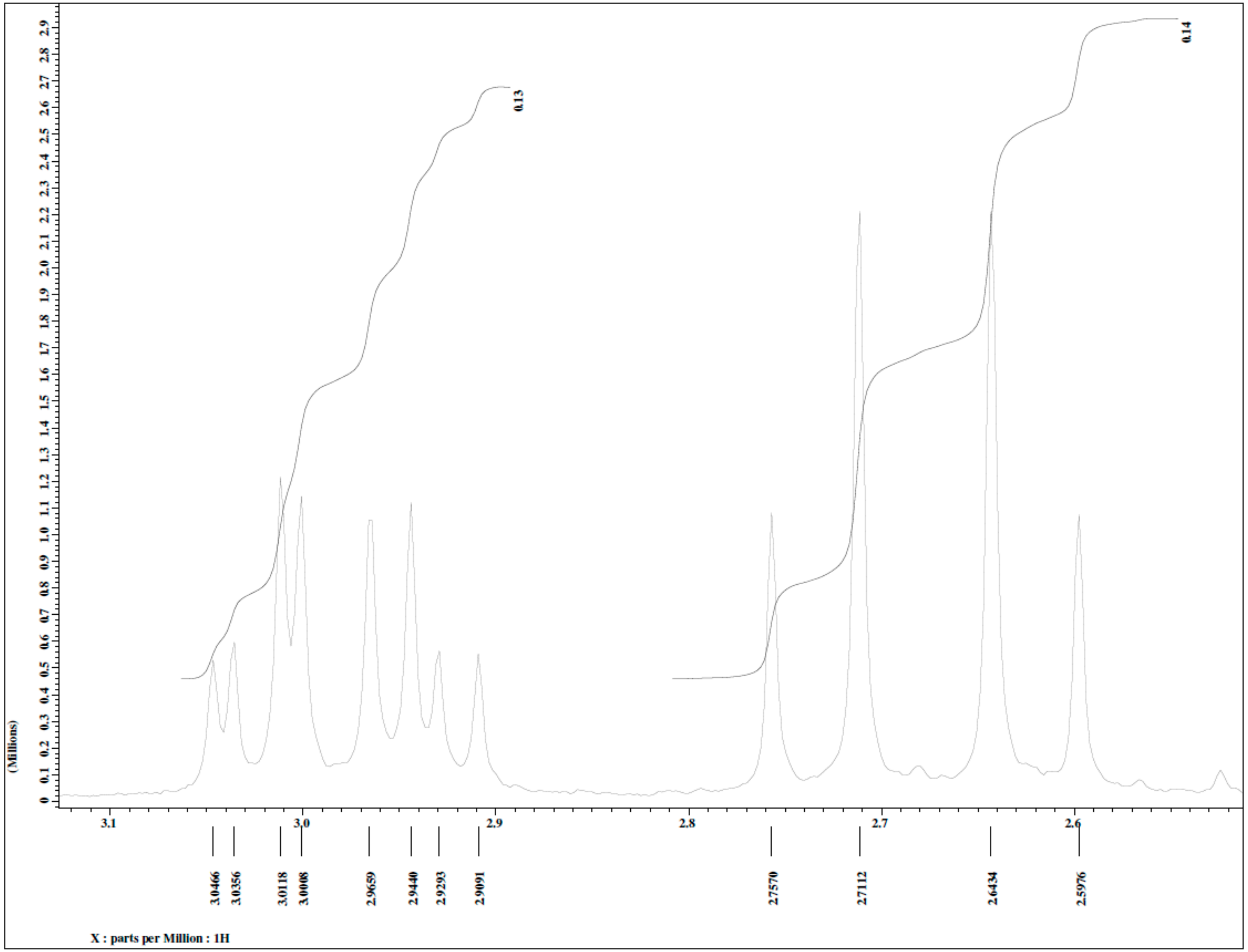

Figure S2. ^{1}H-NMR spectrum of **4b** (Expansion).

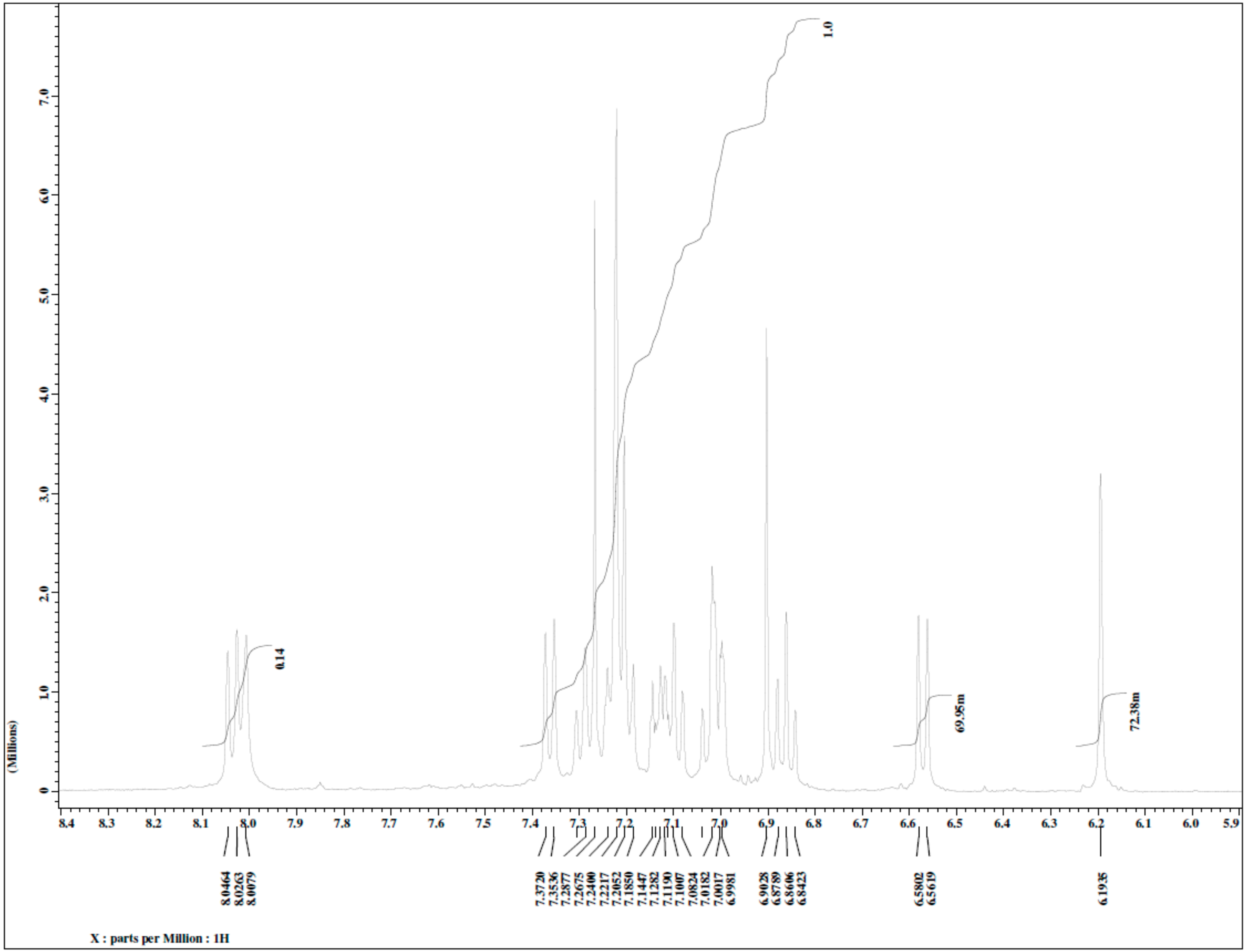

Figure S3. ^{1}H-NMR spectrum of **4b** (Expansion).

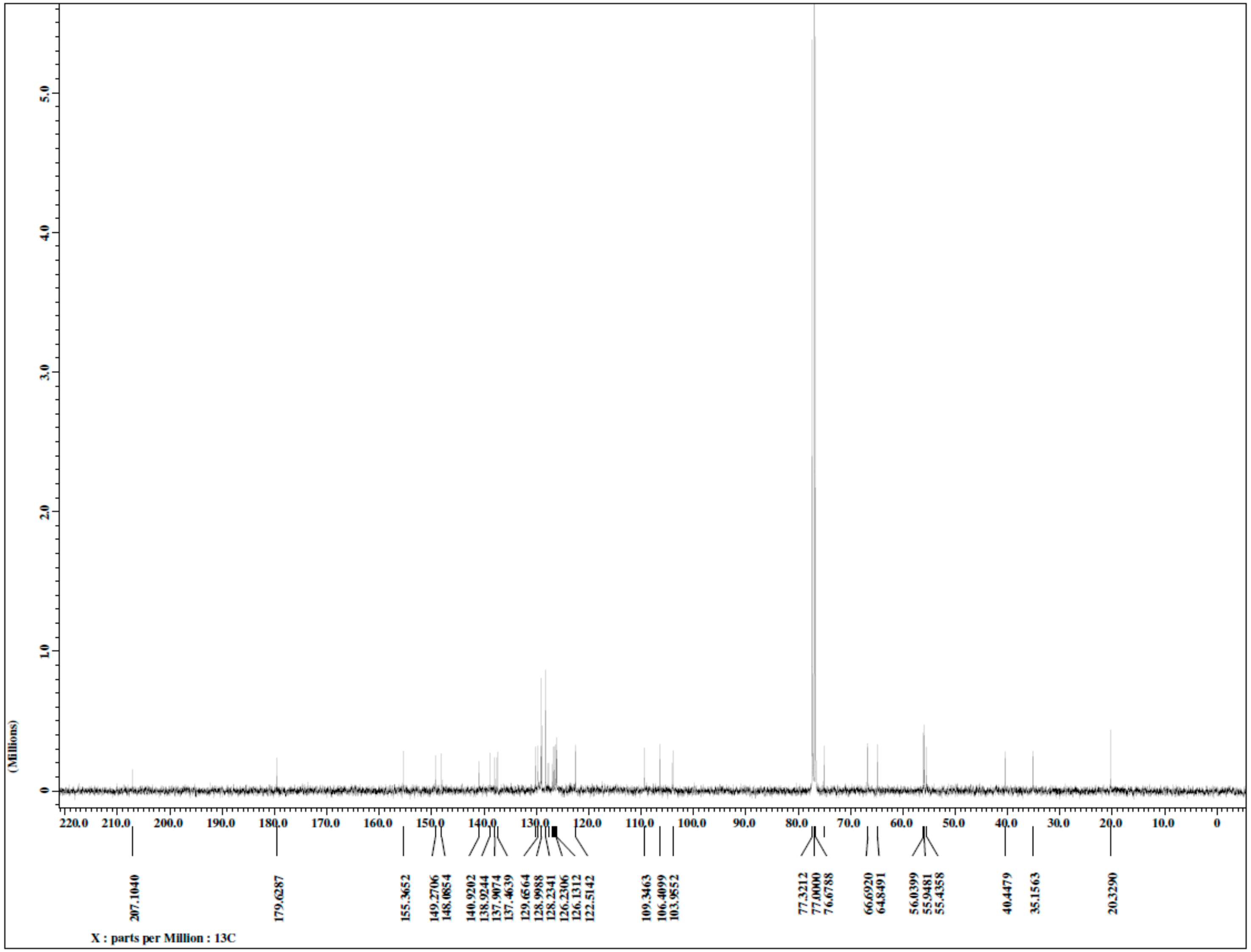

Figure S4. ^{13}C-NMR spectrum of **4b**.

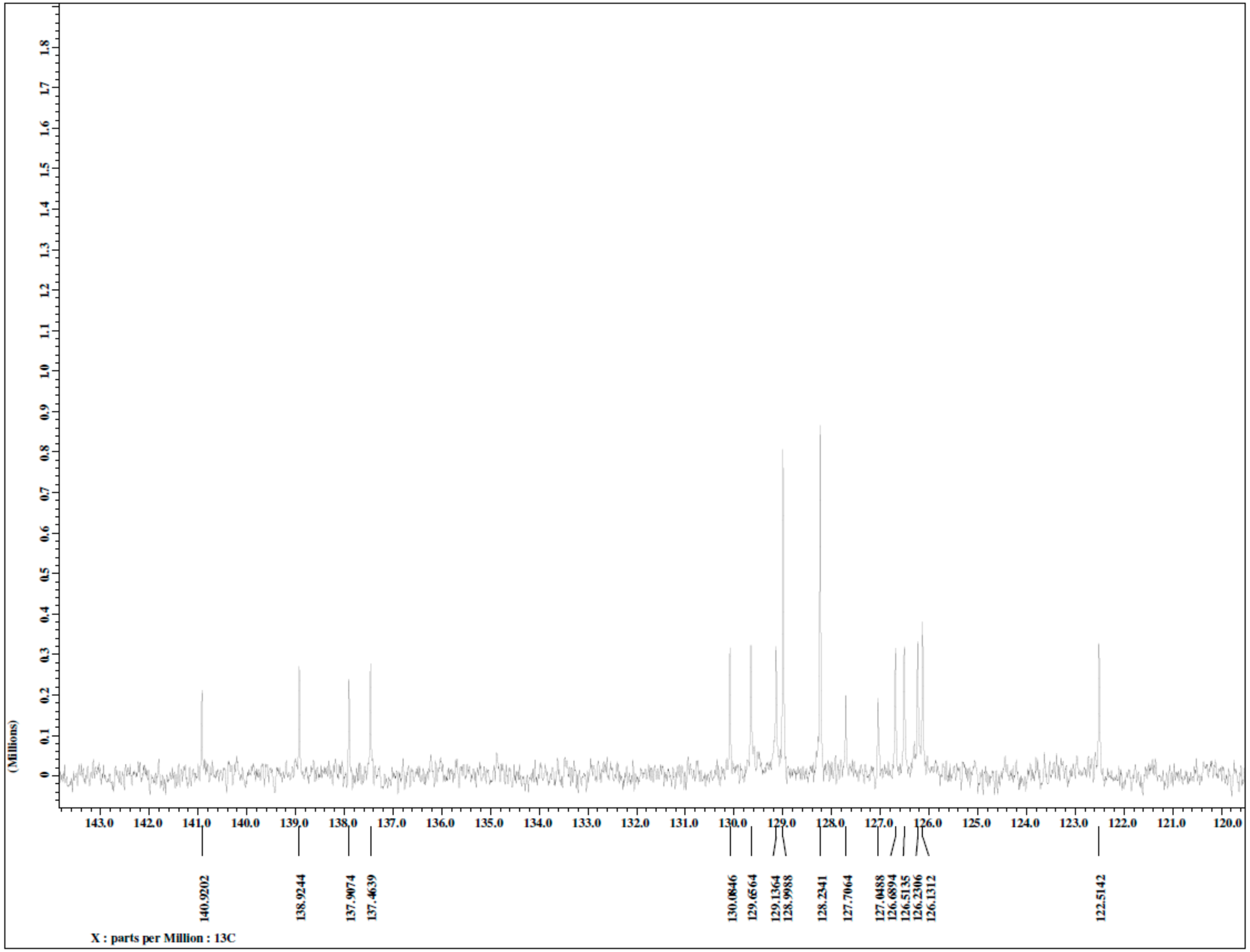

Figure S5. ^{13}C-NMR spectrum of **4b** (Expansion).

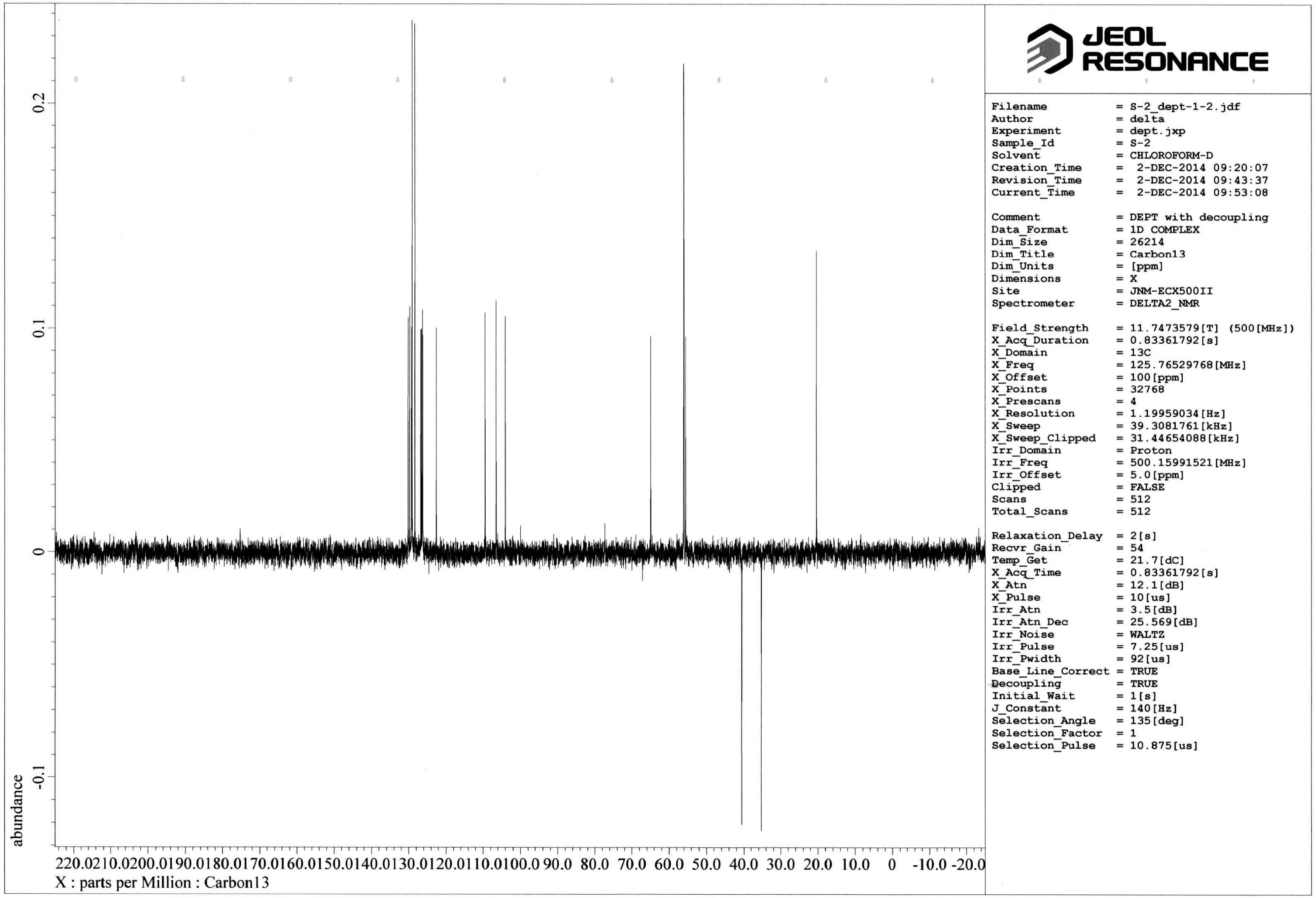

Figure S6. DEPT 135 NMR spectrum of **4b**.

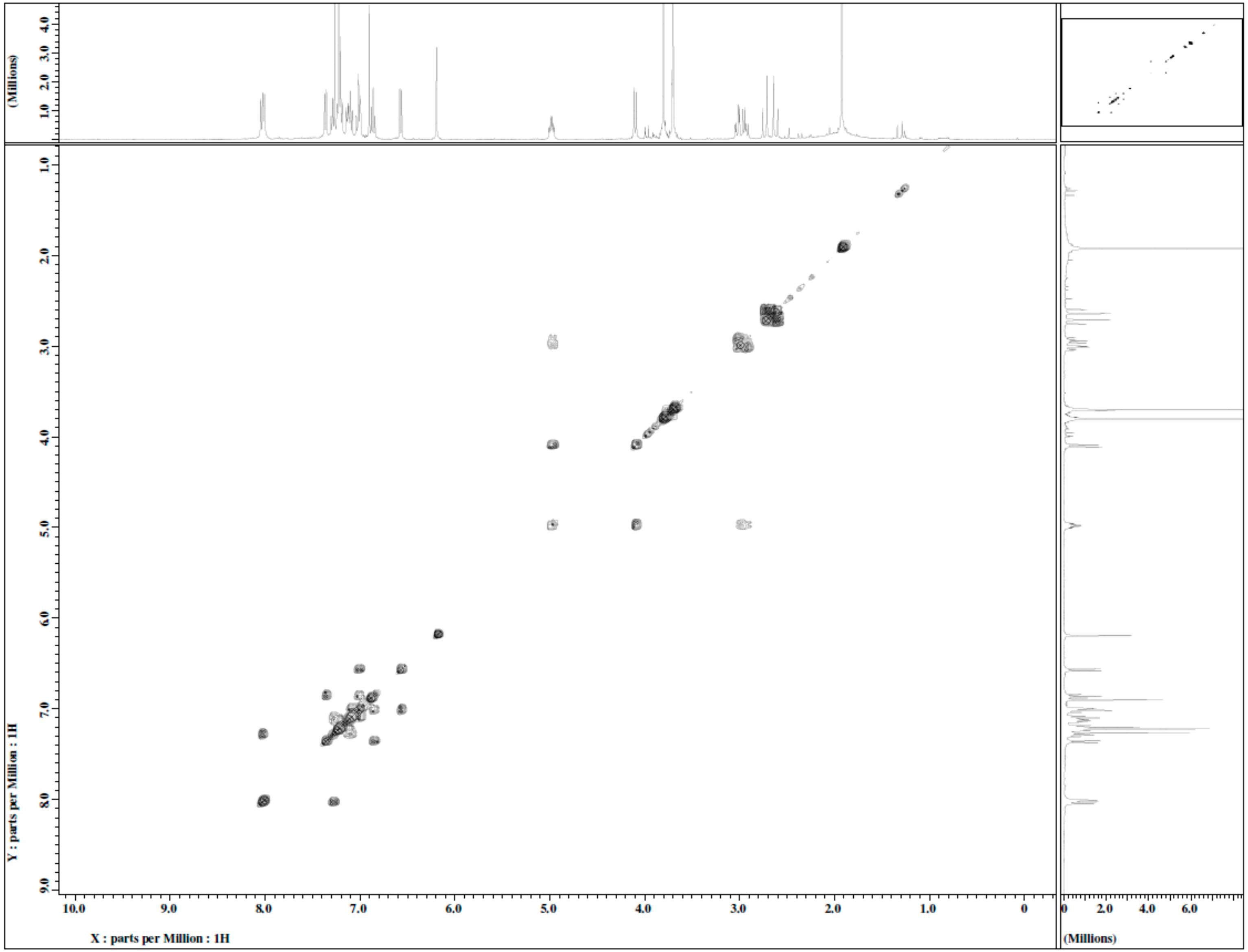

Figure S7. H,H-COSY NMR spectrum of **4b**.

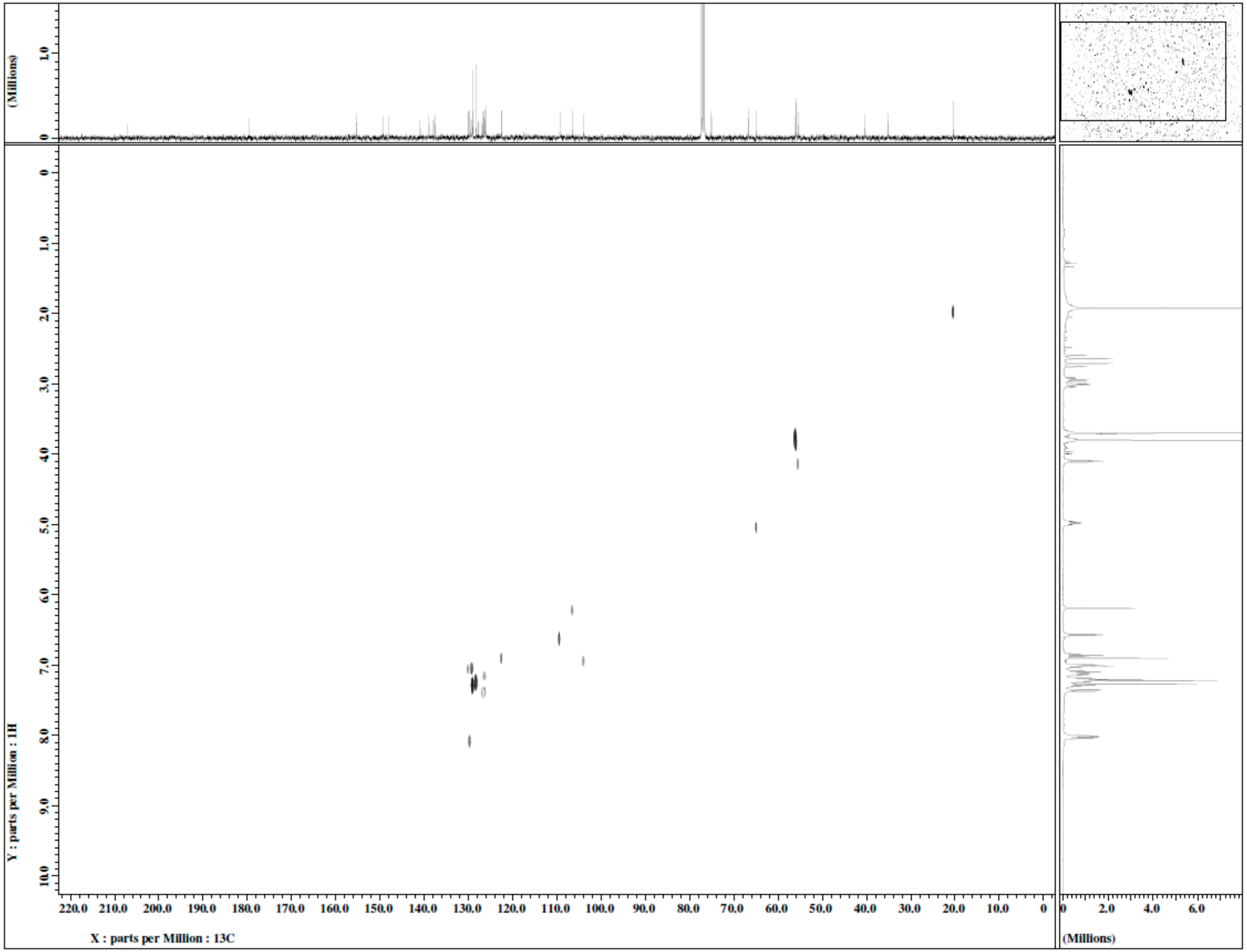

Figure S8. C,H-COSY NMR spectrum of **4b**.

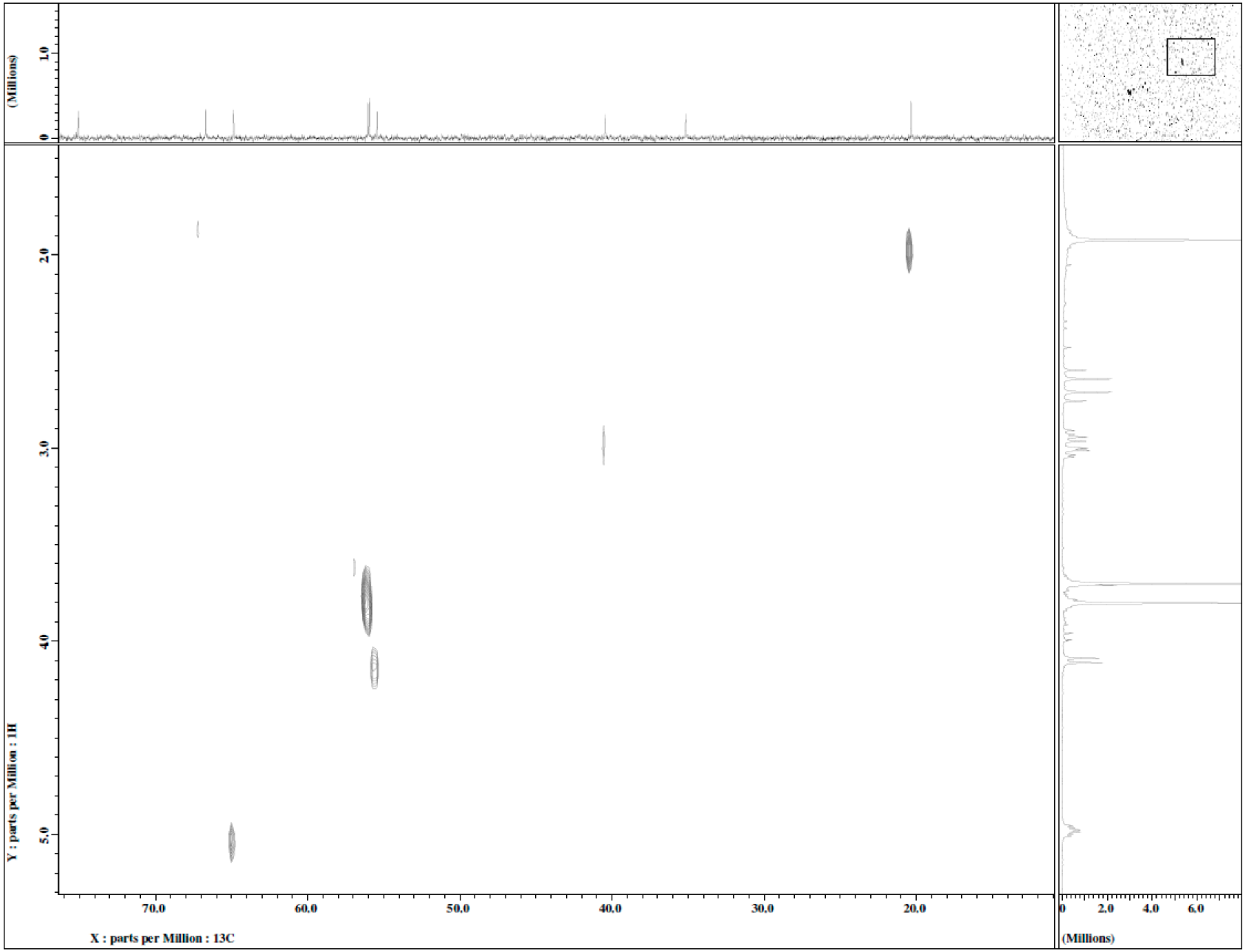

Figure S9. C,H-COSY NMR spectrum of **4b** (Expansion).

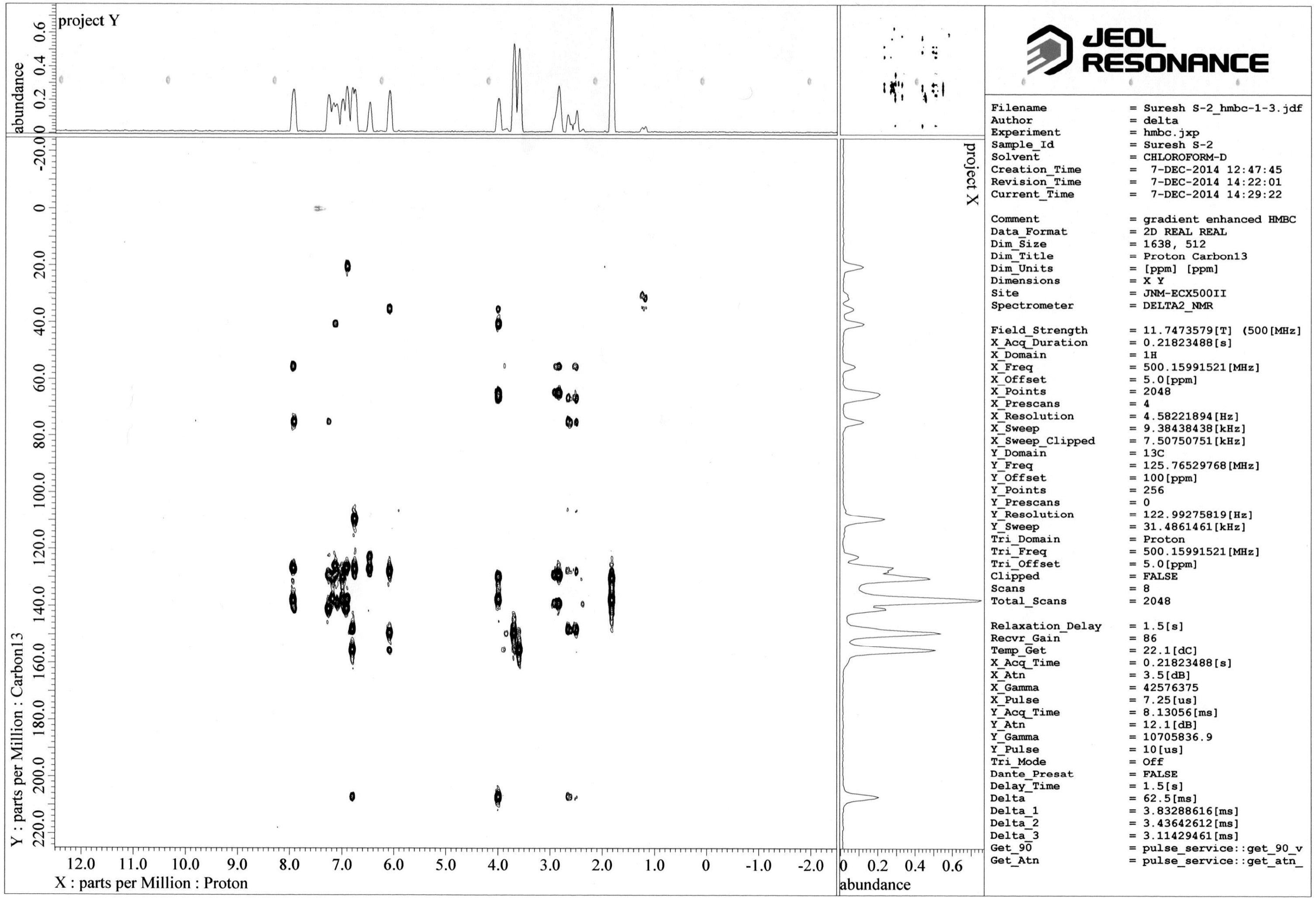

Figure S10. HMBC spectrum of **4b**.

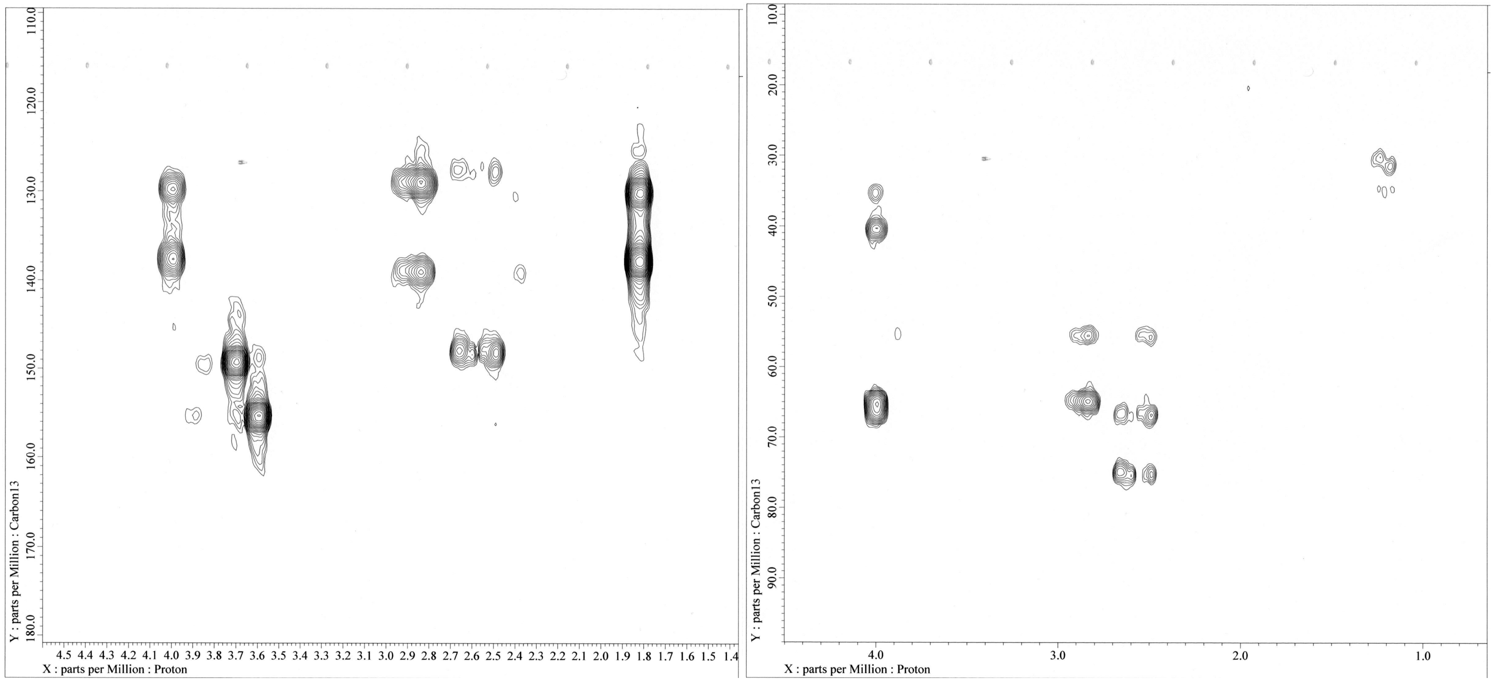

Figure S11. HMBC spectrum of **4b** (Expansion).

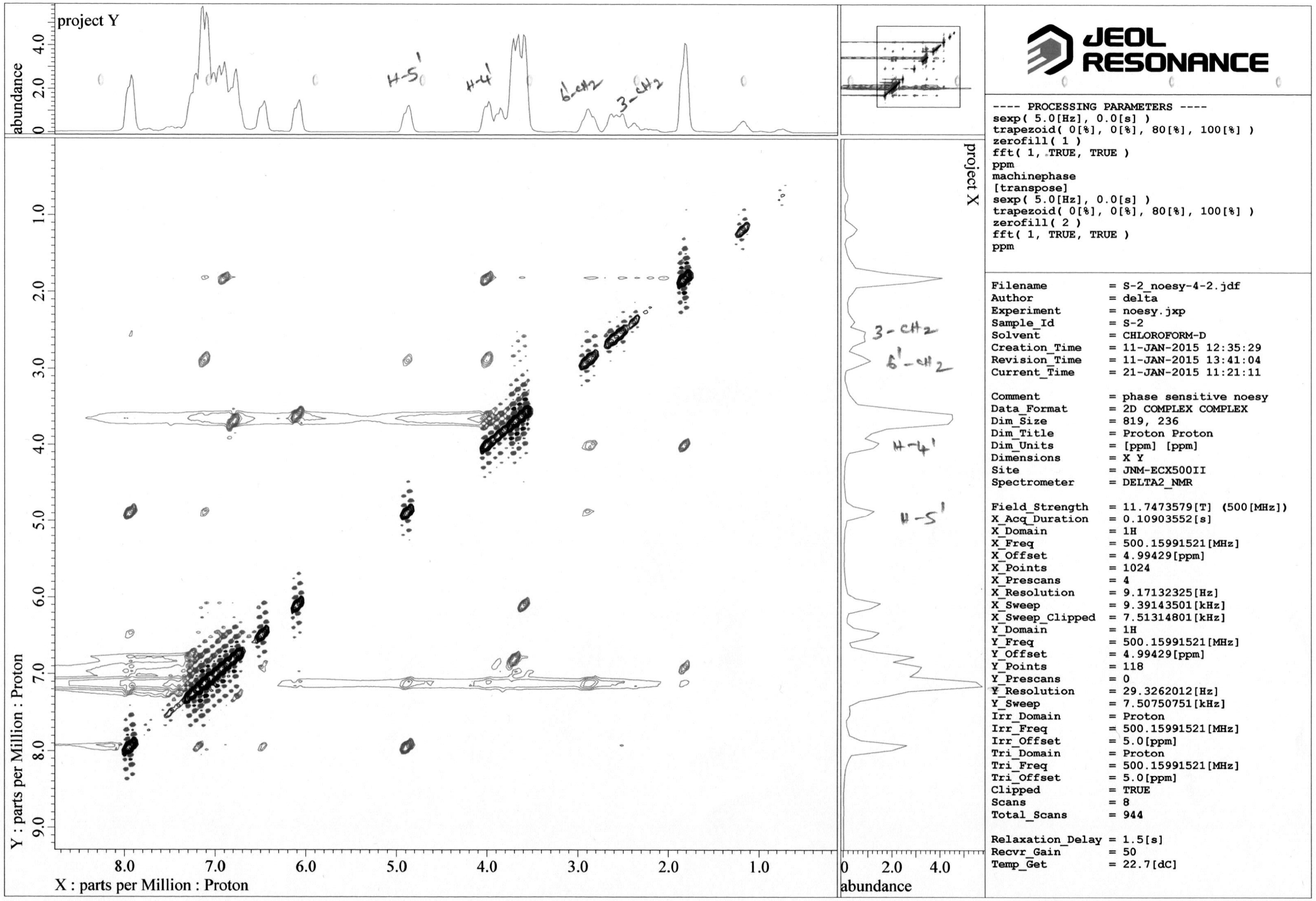

Figure S12. NOESY spectrum of **4b**.

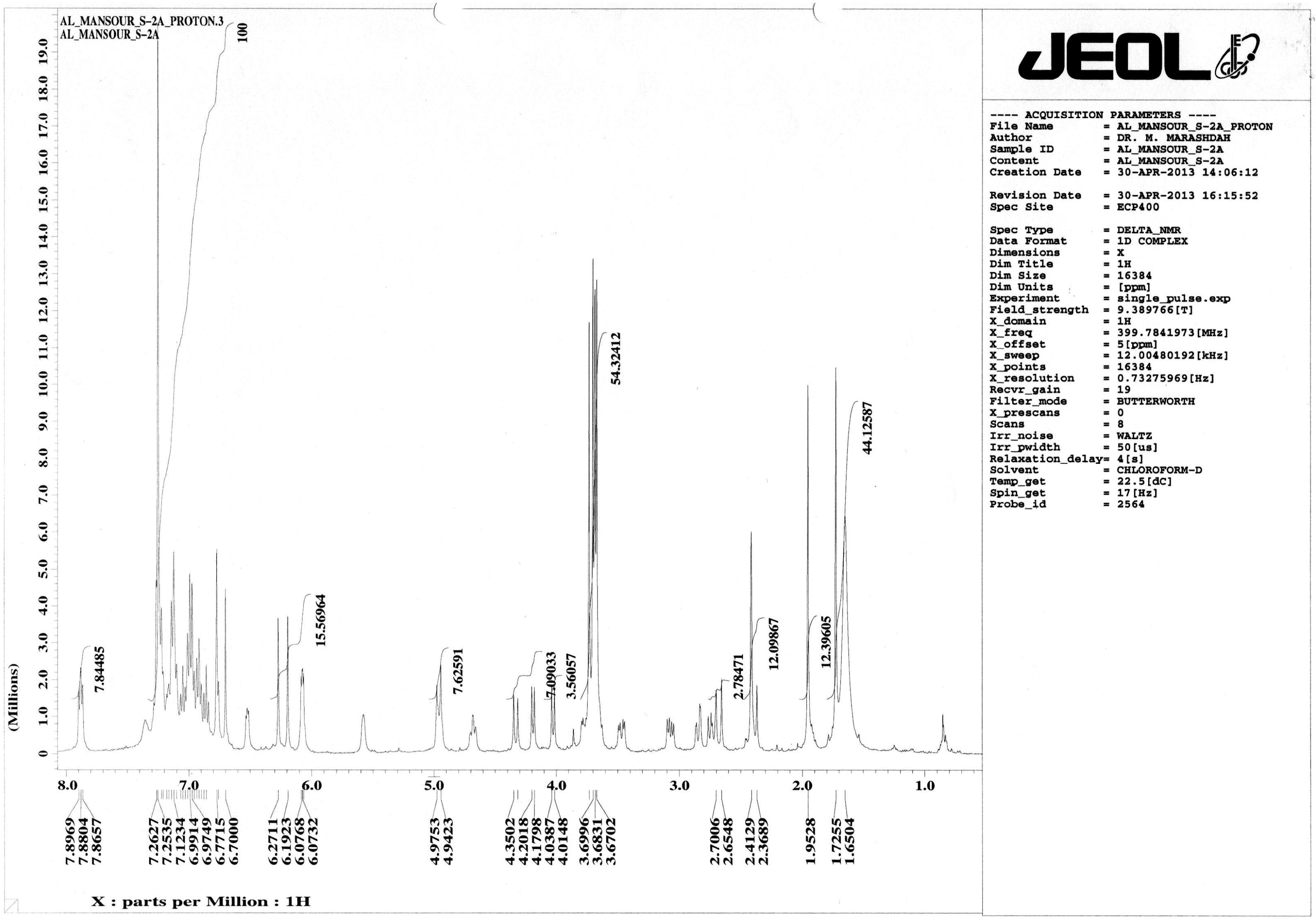

Figure S13. ^{1}H-NMR spectrum of **6b**.

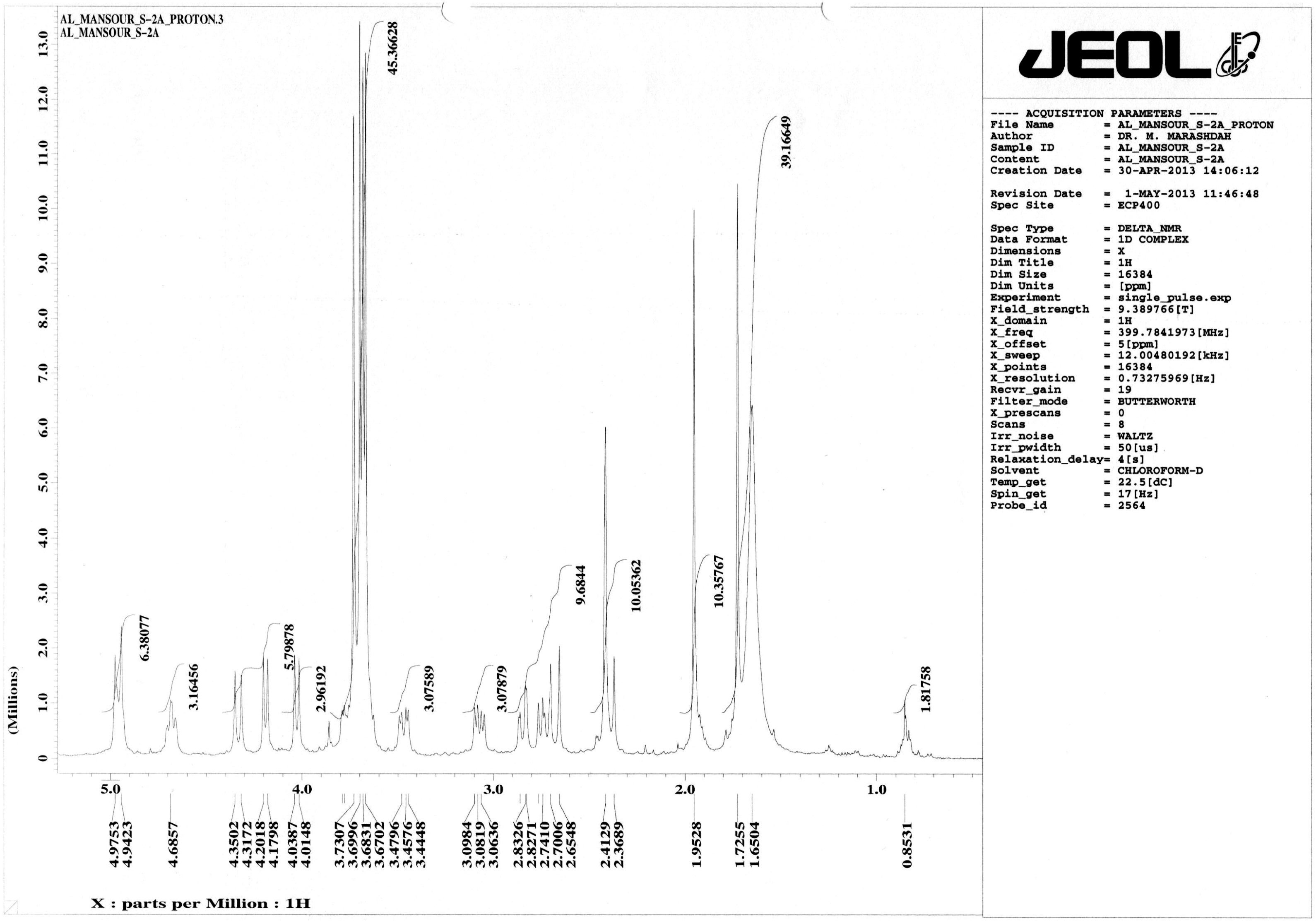

Figure S14. ^{1}H-NMR spectrum of **6b** (Expansion).

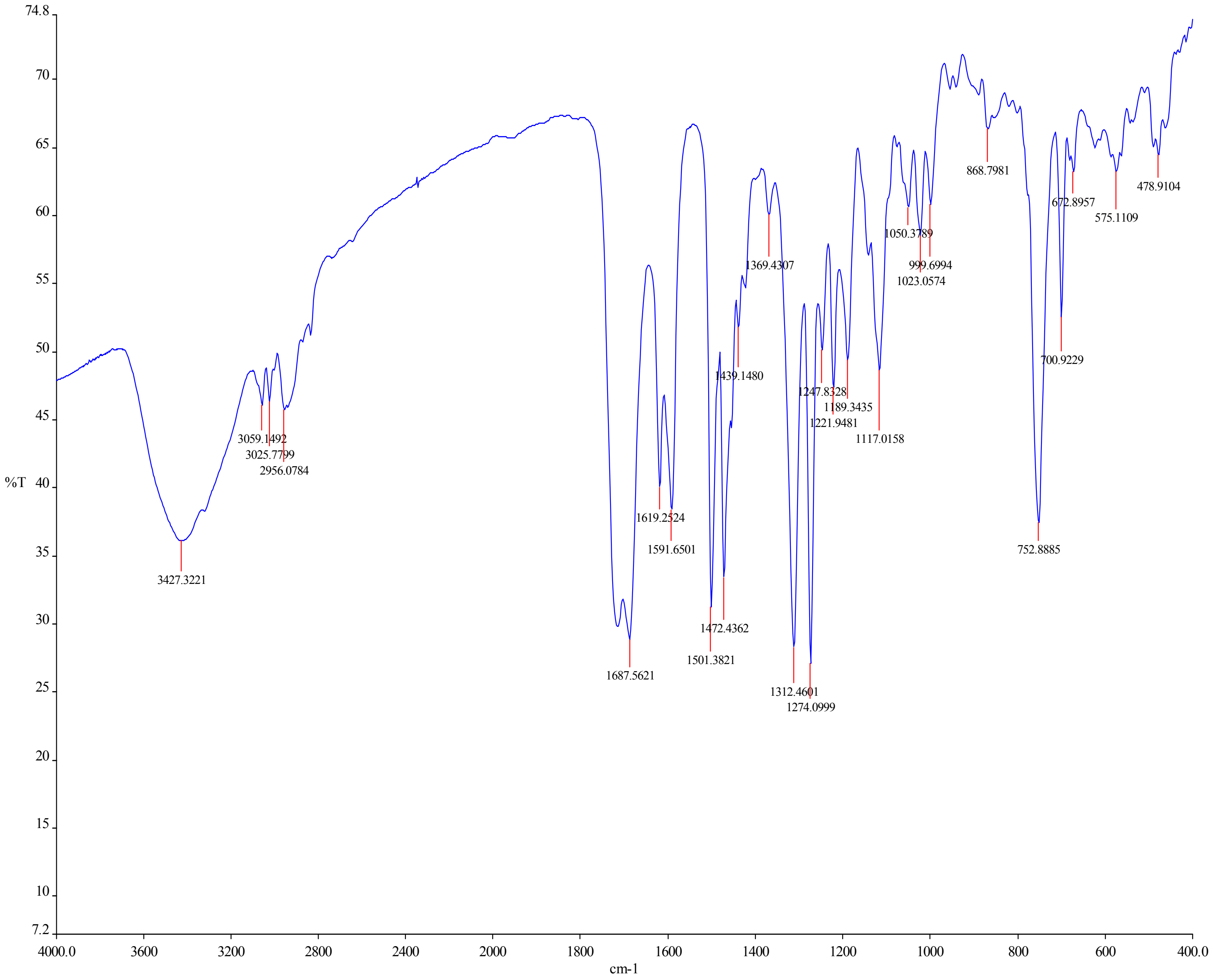

Figure S15. FT-IR spectrum of **4b**.
